# Evaluating Hyperosmolar Water-Soluble Contrast Media for Diagnosing Adhesive Small Bowel Obstruction: A Systematic Review and Meta-Analysis of Randomized Controlled Trials

**DOI:** 10.7759/cureus.95937

**Published:** 2025-11-02

**Authors:** Abdullah H Almeghthawi, May S Alrawithi, Zaher A Alshehri, Najla F Alrwaita, Mansour M Alharbi, Rinad A Albarakati, Mouaz Alothmany, Rama A Babaqi, Abdulrahman F Masoud, Ola A Altwyjri, Lama A Jabbari, Nawaf F Alabbad, Sultan S Alqahtani, Saleh M Aldaqal

**Affiliations:** 1 Department of Surgery, College of Medicine, Taibah University, Madinah, SAU; 2 Department of Surgery, College of Medicine, King Abdulaziz University, Jeddah, SAU; 3 Department of Surgery, College of Medicine, Imam Abdulrahman Bin Faisal University, Dammam, SAU; 4 Department of Surgery, College of Medicine, King Saud Bin Abdulaziz University for Health Sciences, Jeddah, SAU; 5 Department of Surgery, College of Medicine, Umm Al-Qura University, Makkah, SAU; 6 Department of Surgery, College of Medicine, King Khalid University, Abha, SAU; 7 Department of Surgery, College of Medicine, Imam Mohammad Ibn Saud Islamic University, Riyadh, SAU; 8 Department of Radiological Sciences, King Saud Bin Abdulaziz University for Health Sciences, Al Ahsa, SAU; 9 Department of Surgery, King Abdulaziz University Hospital, Jeddah, SAU

**Keywords:** adhesive small bowel obstruction, hospital length of stay, hyperosmolar contrast, systematic review and meta analysis, water-soluble contrast

## Abstract

This systematic review and meta-analysis investigates the use of water-soluble contrast media for diagnosing adhesive small bowel obstruction (ASBO) and predicting the need for surgery. While the potential therapeutic role of hyperosmolar water-soluble contrast (HWSC) is theorized, its efficacy remains uncertain. We assessed the effectiveness of HWSC administration compared to standard nonoperative management in patients with radiologically confirmed ASBO, specifically evaluating hospital length of stay (HLOS) and operative rates. The review was conducted following PRISMA guidelines and registered with PROSPERO. We searched Google Scholar, PubMed, and Web of Science until July 2024 to identify relevant studies. A total of 12 randomized controlled trials with a total of 1150 patients were included. Significant benefit was concluded from the use of hyperosmolar water-soluble contrast (e.g., Gastrografin), demonstrating a reduction in unwarranted prolonged hospitalizations (mean difference - 2.17 days, 95% CI -3.28 to -1.06; p = 0.0001) and a 38% reduction in the need for surgery (RR = 0.63, 95% CI 0.50-0.81; p = 0.0002). No other clinically meaningful differences were observed for secondary outcome measures. The application of HWSC in the management of ASBO appears to shorten HLOS and reduce surgical interventions compared to traditional nonoperative care. Further research is warranted to establish the most effective dosing and scheduling for HWSC administration.

## Introduction and background

Adhesive small bowel obstruction (ASBO) is a common complication of abdominal surgery, representing approximately 60% of all types of intestinal obstruction after surgery [1]. Without early diagnosis and treatment, it can lead to bowel ischemia, perforation, or sepsis, each of which demands urgent medical intervention [2]. Patients who present with features of strangulation or peritonitis need immediate surgical intervention, whereas non-emergency situations may be treated conservatively. Conservative treatment is typically recommended until spontaneous relief of the obstruction becomes apparent and involves fasting, nasogastric tube placement, and intravenous fluids to rehydrate. There is evidence to suggest that conservative management may result in spontaneous relief in nearly 90% of patients [1].

The use of water-soluble contrast media in ASBO has been explored in recent studies, and its diagnostic value in determining the need for surgical intervention has been established [3-5]. While a potential therapeutic role of the contrast medium has been theorized, whether it is effective is contentious [6-8]. If the contrast is observed in the colon within 24 hours, it could indicate a partial obstruction that may be treated using conservative measures. If the contrast agent does not move beyond the small intestine within 24 hours, it can indicate a more serious obstruction that will need prompt surgery [9]. The effectiveness of the hyperosmolar water-soluble contrast (HWSC) agent remains uncertain, primarily due to the variability in the reported data. This variability can result from patient selection, obstruction severity, and administration time. A study by Burge et al. shows that results differ based on the timing of administration and severity of the obstruction, with early administration being beneficial [10]. In contrast, a study by Scotté et al. found no significant reduction in operative rates or hospital length of stay in patients with uncomplicated adhesive small bowel obstruction, a group that typically includes partial or non-strangulated cases [9]. This variation in outcomes across obstruction severity highlights the need to evaluate HWSC’s therapeutic role further.

Nonoperative treatment is recommended in all ASBO patients unless strangulation, peritonitis, or ischemia of the bowel is present. Evidence for the optimal duration of nonoperative management is lacking; however, most studies suggest that a 72-hour period is safe and appropriate [1]. Although encouraging results have been observed, gaps remain in the understanding of HWSC utilization.

This systematic review and meta-analysis determined the efficacy of HWSC, especially Gastrografin, in radiographically confirmed ASBO, compared with that of standard nonoperative management. The primary outcome variables were hospital length of stay (HLOS) and operative rates. This review examined pooled data from randomized controlled trials (RCTs) on ASBO management and evaluated HWSC to provide clearer insights.

## Review

Methods

We registered the protocol in PROSPERO with ID: CRD42024570665. Ethics board approval was not required owing to the study type. In order to minimize potential bias in the included studies, we followed the Preferred Reporting Items for Systematic Reviews and Meta-Analyses (PRISMA) guidelines [11].

Inclusion Criteria

The included studies met five criteria: (1) patients with ASBO confirmed by radiological imaging; (2) HWSC administration; (3) comparison with placebo or standard nonoperative management; (4) reporting of at least one of the primary outcomes (HLOS or operative rates), and secondary outcomes, including time to resolution of symptoms, time to surgery, rates of bowel resection, mortality rates, and complication rates; and (5) RCTs.

Exclusion Criteria

Exclusion criteria were as follows: (1) the study sample included cases of non-ASBO, malignant bowel obstruction, prior abdominal radiation, recent abdominal surgery within six weeks, or hernia-causing obstruction; (2) the study included the use of non-hyperosmolar or low-osmolarity contrast agents or any other non-standardized intervention; (3) studies without a control group or using barium sulfate as the contrast agent; (4) studies not reporting outcomes of interest; and (5) non-randomized controlled studies.

Search Strategy

The PubMed, Web of Science, and Google Scholar databases were systematically searched in July 2024. We used keywords (water-soluble contrast or hyperosmolar contrast or Gastrografin) and (small bowel obstruction or intestinal obstruction or SBO or IO). Additionally, we evaluated studies based on the population, intervention, comparison, and outcome (PICO) framework [12]. The core concepts included terms for "adhesive small bowel obstruction" and "hyperosmolar water-soluble contrast." The full, reproducible search strings with all applicable Boolean operators and filters are provided in the Appendix. No time limits were applied; however, only studies published in English were evaluated. 

Study Selection

**Figure 1:**
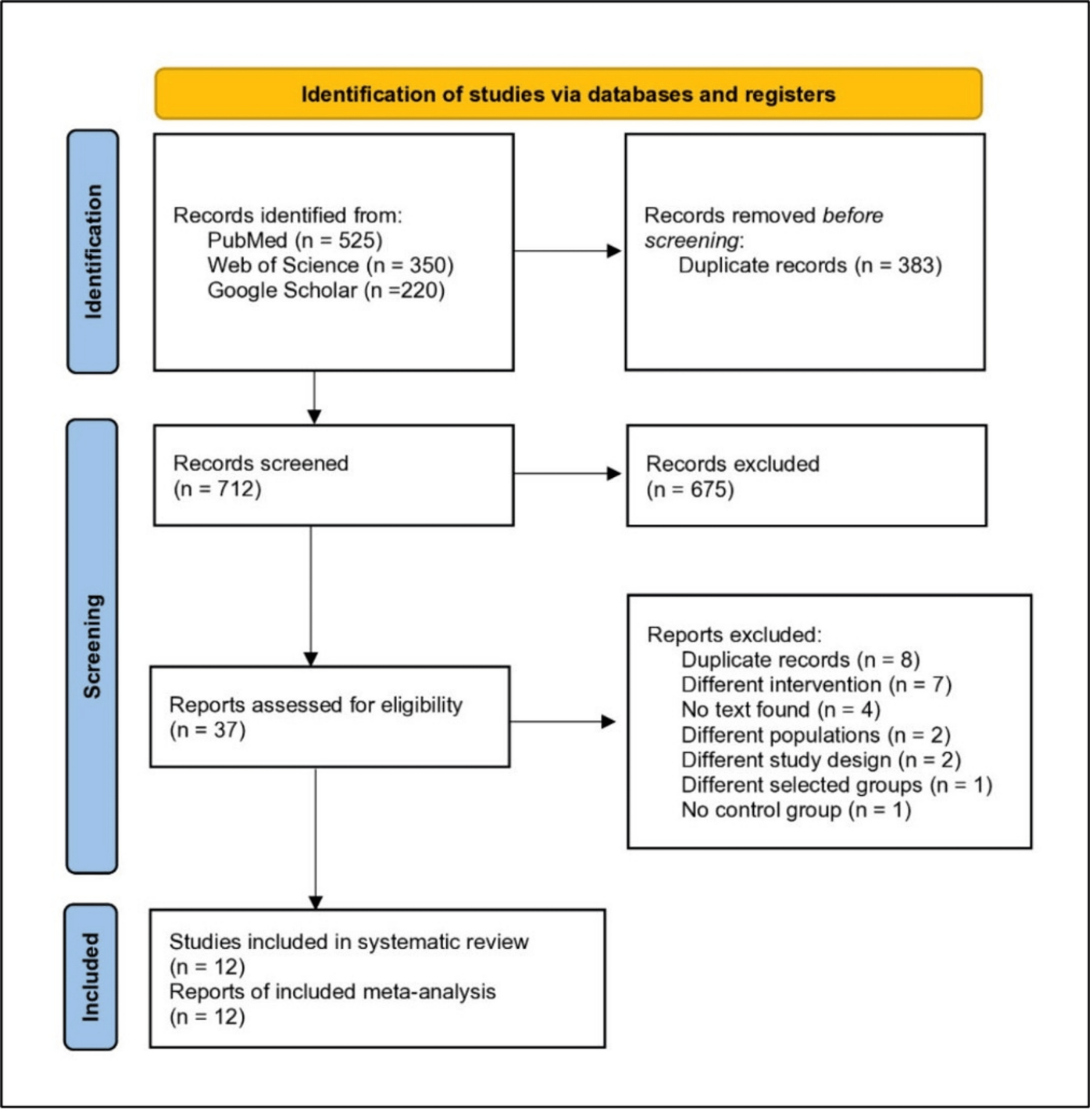
PRISMA flowchart.

Two reviewers independently screened the papers by title and abstract through the Rayyan web and mobile applications. Subsequently, two independent reviewers examined the full texts with a third reviewer who helped resolve conflicts alongside a consultant who assessed the clinical relevance of each study. The screening process for eligibility is shown in Figure 1 [13].

Outcome Measures

The primary outcomes derived from all 22 variables were used to evaluate the predictive role and accuracy of HWSC regarding surgical requirements. The primary outcome was used to measure the correlation with many variables, including demographic and other clinically relevant variables.

Data Extraction and Quality Assessment

Data extraction was performed by two independent reviewers into a predefined Excel sheet following the full-text screening. Any discrepancies between the reviewers were resolved through discussion or by consulting a third researcher. The reviewers extracted data for the following 22 variables: (1) total number of patients, (2) age range, (3) mean age (years), (4) standard deviation of age, (5) number of male patients, (6) number of female patients, (7) number of comorbidities, (8) number of patients smoking, (9) follow-up duration in months, (10) intervention type, (11) comparison group, (12) treatment duration, (13) HLOS, (14) operative rates, (15) time to resolution of symptoms, (16) time to surgery, (17) rates of bowel resection, (18) mortality rates, (19) complication rates, (20) measurement tools, (21) time points of measurement, (22) clinical recommendations or conclusions, and (23) significant outcomes and p-values. The finalized data sheet was double-checked to avoid duplication.

The included studies were assessed for potential RoB and quality. The Cochrane RoB tool for randomized trials (RoB 2) was used to assess the risk of bias in the included studies. Two authors (OA and LJ) independently performed the RoB assessments, which were categorized into seven domains: selection, performance, detection, attrition, reporting, other, and overall RoB [14]. Each study was evaluated for these biases and categorized as having low RoB, high RoB, or unclear RoB. Subsequently, the overall RoB was determined: studies were considered low RoB if all the domains showed low RoB; high RoB if any domain had high RoB; or unclear RoB if at least one domain was unclear.

Statistical Analysis

**Figure 2:**
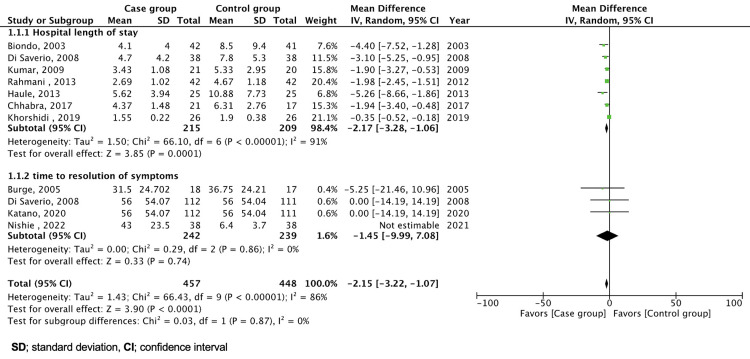
Forest plot of hospital length of stay and time to resolution of symptoms. Burge et al., 2005 [10], Biondo et al., 2003 [15], Di Saverio et al., 2008 [16], Kumar et al., 2009 [17], Rahmani et al., 2013 [18], Haule et al., 2013 [19], Chhabra et al., 2017 [20], Khorshidi et al., 2019 [21], Katano et al., 2020 [22], Nishie et al., 2022 [23]

**Figure 3:**
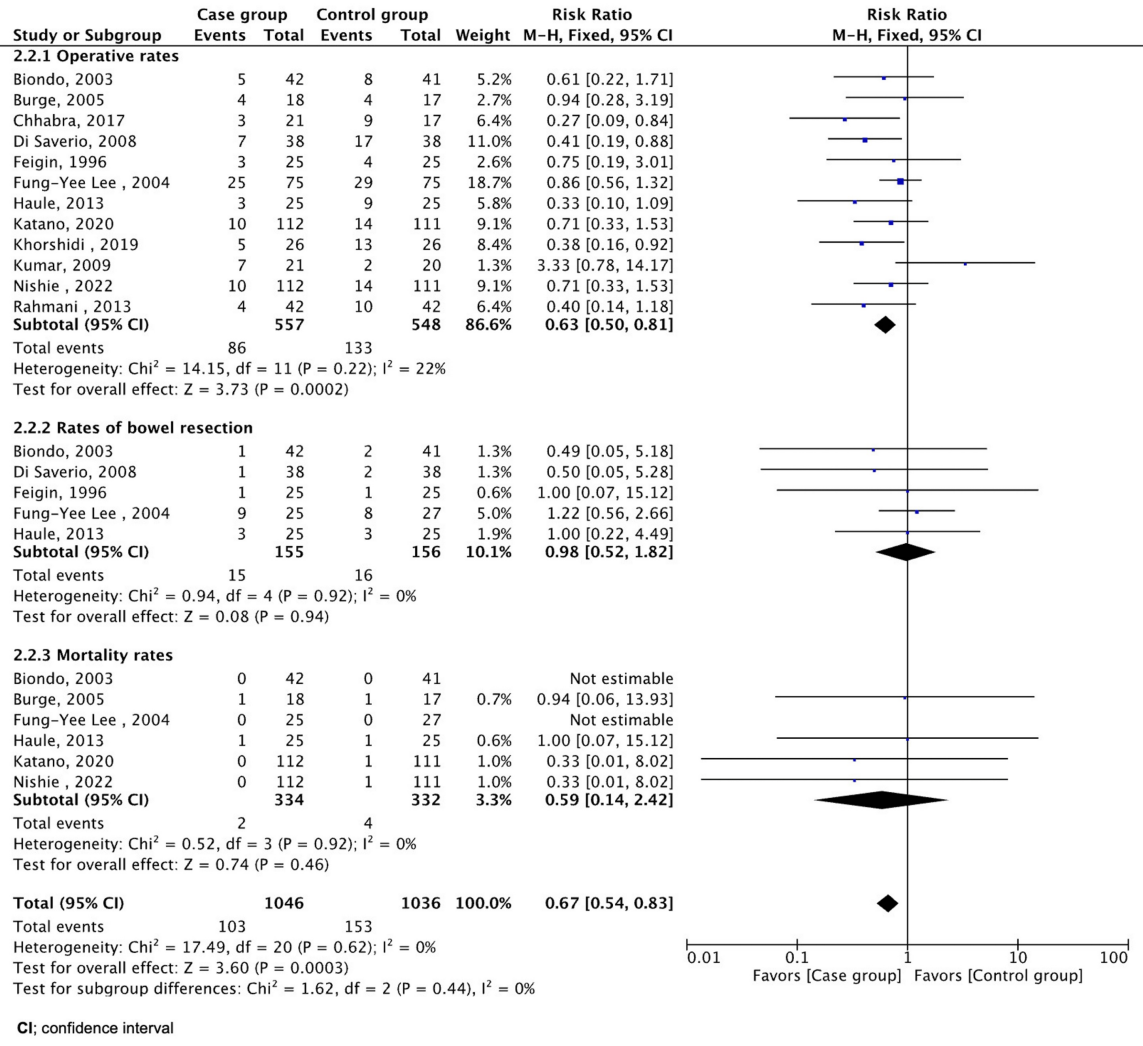
Forest plot of rates of operation, bowel resection, and mortality. Feigin et al., 1996 [7], Burge et al., 2005 [10], Biondo et al., 2003 [15], Di Saverio et al., 2008 [16], Kumar et al., 2009 [17], Rahmani et al., 2013 [18], Haule et al., 2013 [19], Chhabra et al., 2017 [20], Khorshidi et al., 2019 [21], Katano et al., 2020 [22], Nishie et al., 2022 [23], Lee et al., 2004 [24]

We performed statistical analysis using RevMan 5.4 software (The Cochrane Collaboration, London, UK). The analyses were divided into primary and secondary outcomes. We reported the mean with standard deviation and a 95% confidence interval (CI) for continuous variables. Studies that did not report the values of interest were excluded. Dichotomous outcomes were reported as risk ratios (RR) with a 95% CI, except for studies that lacked relevant data. Heterogeneity was assessed. The variables were collected and entered for analysis from included articles, considering compatibility, and where missing variables from a specific study were excluded in the analysis for that specific variable, as shown in Figures 2-3. No method of data conversion was used. The results are summarized in forest plots, tables, and flow charts.

Results

Study Selection

After screening 712 abstracts, only 37 publications were eligible for full-text screening. After reviewing the eligible studies, two studies had non-eligible study designs [25,26], seven studies had different interventions [27-33], eight studies were duplicates of other included studies [15,19], and full text was not available for four studies [6,8,34,35]. One study did not have a control group [4], and two studies had different populations [36,37]; one study had different control and intervention groups [38]. Only 12 studies evaluated HWSC in ASBO [7,10,15-24] (Figure 1) [13].

Characteristics of Included Studies

The studies were all RCTs with geographical distribution across several countries, including Japan, India, and Iran. Two studies (16.7%) each were identified in these countries. One study (8.3%) was found in the remaining countries, including Italy, Spain, Uganda, Hong Kong, New Zealand, and Israel. The total number of patients with postoperative bowel obstruction in all the studies was 1150. Patients' ages in all the studies were between 5 and 91 years (Table 1). 

**Table 1:** Characteristics of included studies. Abbreviations: RCT, randomized controlled trial; TTS, time to surgery; HLOS, hospital length of stay; OR, odds ratio; TTRS, time to resolution of symptoms; NGT-G, nasogastric tube group; LT, long tube.

Study	Publication year	Study Design	Study Country	Sample size	Case group	Control group	Age range	Comorbidities	Outcomes, P-values
Biondo et al. [15]	2003	RCT	Spain	90	44 (Male, 33; Female, 11)	46 (Male, 29; Female, 18)	Control (23-91), Case (24-86)	Control, 29; Case, 29	TTS: P= 0.002, HLOS: P< 0.001
Di Saverio et al. [16]	2008	RCT	Italy	76	36 (Male, 18; Female, 18)	40 (Male, 20; Female, 20)	Both groups, (22-85)	N/A	OR: P<0.02, TTRS : P<0.01, HLOS: P< 0.05
Katano et al. [22]	2020	RCT	Japan	223	112 (Male, 57; Female, 55)	111 (Male, 69; Female, 42)	Control (61-82), Case (64-79)	N/A	Non-inferiority: P =0.00002923 for the non-surgery rate of NGT-G compared to LT
Haule et al. [19]	2013	RCT	Uganda	50	25 (Male, 18; Female, 7)	25 (Male, 17; Female, 8)	N/A	N/A	HLOS: P = 0.04
Lee et al. [24]	2004	RCT	Hong Kong	150	75 (Male, 52; Female, 23)	75 (Male, 45; Female, 30)	Control (17-87), Case(16-90)	Control, 24; Case, 20	TTRS: P = 0.023
Rahmani et al. [18]	2013	RCT	Iran	84	42 (Male, 27; Female, 15)	42 (Male, 23; Female, 19)	Both groups ( ≥18)	N/A	HLOS: P = 0.025
Nishie et al. [23]	2022	RCT	Japan	223	112 (Male, 57; Female, 55)	111 (Male, 69; Female, 42)	Both groups (20-90)	N/A	Preoperative observation period: P = 0.014
Khorshidi et al [21]	2019	RCT	Iran	52	26 (Male, 16; Female, 10)	26 (Male, 18; Female, 8)	Control (38-75), Case, (37-81)	N/A	HLOS: P = 0.00, OR: P = 0.07
Burge et al. [10]	2005	RCT	New Zealand	35	18 (gender not specified)	17 (gender not specified)	Control (41-99), Case (34-97)	N/A	HLOS, P = 0.342
Kumar et al. [17]	2009	RCT	India	41	21 (Male, 17; Female, 4)	20 (Male, 14; Female, 6)	Control (19-80), Case (19-70)	N/A	Improvement of symptoms p=0.569
Feigin et al. [7]	1996	RCT	Israel	50	25 (gender not specified)	25 (gender not specified)	Both groups (5-88)	N/A	Number of patients successfully treated (P=0.04)
Chhabra et al. [20]	2017	RCT	India	38	21 (Male, 11; Female, 10)	17 (Male, 10; Female, 7)	Control (15-81), Case (14-72)	N/A	Mean HLOS: (P=0.004)

Patients in the intervention groups received Gastrografin orally or via nasogastric tube. Study duration varied in all studies. Control groups received standard conservative treatment, saline, or long tube decompression (Table 2).

**Table 2:** Intervention details. Abbreviations: NGT, nasogastric tube; NGT-G, nasogastric tube group; h, hours; N/A, not applicable.

Study	Type of intervention	Comparison group	Duration of treatment
Biondo et al. [15]	Gastrografin through the NGT-G	Standard treatment without Gastrografin	After 24 hours, if the Gastrografin failed to reach the colon, the patient underwent laparotomy
Di Saverio et al. [16]	Gastrografin meal	Traditional conservative treatment	Differs based on the response to Gastrografin (36 and 72 hours)
Katano et al. [22]	Intubation of NGT-G	Long tube	48-72 h
Haule et al. [19]	Gastrografin administration	Standard conservative treatment group	1-14 days
Lee et al. [24]	Water-soluble contrast follow-through using 76% Urografin	Control group without contrast follow-through	N/A
Rahmani et al. [18]	100 mL Gastrografin was administered through NGT	Conventional treatment	N/A
Nishie et al. [23]	Administration of NGT-G	Long tube	N/A
Khorshidi et al. [21]	100 mL of oral Gastrografin solution given through NGT	Conventional treatment (100 mL of 0.9% saline solution)	Conservative treatment was assessed over a period of 48 hours. Patients were monitored for improvement in symptoms or signs of resolution during this time.
Burge et al. [10]	100 mL of Gastrografin	Placebo (isotonic saline)	N/A
Kumar et al. [17]	60 mL of oral Gastrografin	Conventional treatment	Erect and supine abdominal radiographs were repeated after 12 hours and then subsequently when necessary.
Feigin et al. [7]	Standard treatment with 100 mL of meglumine ioxitalamate	Standard treatment	Up to 5 days if no improvement surgery was performed
Chhabra et al. [20]	60 mL of Gastrografin admixed with 40 mL of normal saline	100 mL of normal saline	48 to 72 hours

Assessment of Risk of Bias

Eight studies [7,17-23] were rated as having a high risk of bias. These studies had at least one domain marked as high risk, such as detection or attrition bias, which may compromise the validity of their findings. Five studies [10,15,16,20,24] were classified as having an unclear risk of bias. These studies had several domains marked as unclear, making it difficult to fully assess the reliability of their results. Table 3 provides a detailed summary of the risk of bias assessment for all included studies.

**Table 3:** Risk of Bias Assessment. Abbreviations: RCT, randomized controlled trial; L, low risk of bias; U, unclear risk of bias; H = high risk of bias

Authors (Year)	Study Type	Selection Bias	Detection Bias	Attrition Bias	Reporting Bias	Other Bias	Overall RoB
Rahmani et al., 2013 [18]	RCT	L	U	U	U	U	H
Biondo et al., 2003 [15]	RCT	U	U	U	L	L	U
Di Saverio et al., 2008 [16]	RCT	L	L	U	L	L	U
Katano et al., 2020 [22]	RCT	L	L	H	L	L	H
Haule et al., 2013 [19]	RCT	L	L	H	L	L	H
Lee et al., 2004 [24]	RCT	L	L	U	L	L	U
Nishie et al., 2022 [23]	RCT	L	H	U	L	L	H
Khorshidi et al., 2019 [21]	RCT	L	H	U	L	L	H
Burge et al., 2005 [10]	RCT	L	L	U	U	L	U
Kumar et al., 2009 [17]	RCT	L	H	U	L	L	H
Feigin et al., 1996 [7]	RCT	L	H	U	L	L	H
Chhabra et al., 2017 [20]	RCT	L	U	U	L	L	U

Outcomes

The results of a meta-analysis of HLOS are shown in Figure 2. The combined analysis, utilizing seven studies, indicates a decrease in HLOS when treated with HWSC versus conservative treatment. The total mean difference (MD) was -2.17 days (95% CI: -3.28, -1.06, P = 0.0001) in favor of HWSC. Despite high heterogeneity (I² = 91%), the data suggest that HWSC can be linked with shorter hospital stays.

Four studies reported data on the time to resolution of symptoms [10,16,22,23]. The heterogeneity between the studies was high. However, after removing the study by Nishe et al., heterogeneity decreased to 0% (I2 = 0%), resulting in an overall MD of -1.45 (95% CI: -9.99 to 7.08) (Figure 2) [23].

The meta-analysis of operative rates compares the effect of Gastrografin treatment with that of a control intervention (Figure 3). Pooled analysis revealed a reduction in operative rates among patients who received Gastrografin. The RR was 0.63 (95% CI: 0.50-0.81, P = 0.0002), and heterogeneity was low (I² = 22%).

A meta-analysis of five trials was conducted to determine how commonly patients treated with Gastrografin underwent bowel resection compared with the control group [7,15,16,19,24]. The pooled RR was 0.98 (95% CI: 0.52, 1.82), indicating no significant difference between the groups (Figure 3). No significant heterogeneity was observed among the included studies.

The pooled RR for mortality in six trials was 0.59 (95% CI: 0.14, 2.42) (Figure 3) [10,15,19,22-24]. The heterogeneity among the studies was low. 

Rates of Complications and Time to Surgery

We included this outcome in the study to emphasize the effectiveness of Gastrografin. However, the meta-analysis was not performed due to the lack of data. For example, only one study reported the time to surgery [15], and only two studies provided the complication rate [16,22].

Discussion**

This systematic review and meta-analysis evaluated the efficacy of hyperosmolar HWSC in the management of ASBO. The findings indicate that HWSC serves as a valuable therapeutic adjuvant in conservative ASBO management, contributing to a statistically significant reduction in both HLOS and operative rates. Reducing HLOS is a critical goal, as prolonged hospitalization increases healthcare costs and patient morbidity. The observed reduction in operative rates suggests that HWSC may aid in clinical decision-making by helping to distinguish patients who will respond to conservative management from those who require surgical intervention. However, HWSC did not demonstrate a significant effect on secondary outcomes, including mortality, bowel resection rates, and time to resolution of symptoms. The benefit of HWSC appears to be most pronounced in cases of partial rather than complete obstruction, where surgery remains the definitive treatment [39]. This underscores the importance of careful patient selection.

Our findings align with prior research indicating that HWSC reduces the need for surgery. However, the impact on HLOS has been inconsistent across the literature. For instance, a systematic review from a Canadian center found no significant difference in HLOS [40], whereas our pooled analysis demonstrates a significant reduction. These discrepancies may be attributed to variability in patient selection, timing of HWSC administration, and methodological differences among the included trials [39]. The significant reduction in operative rates and HLOS observed in our analysis supports the integration of HWSC into standardized clinical guidelines for ASBO to minimize unnecessary surgeries and improve healthcare resource utilization.

The timing of HWSC administration is a crucial factor influencing its effectiveness. Previous meta-analyses have indicated that early administration within the first 24 hours may enhance its therapeutic effect, while delayed administration diminishes its potential [39]. This aligns with the World Society of Emergency Surgery Bologna Guidelines 2018, which advocate for early nonoperative management in selected ASBO cases [1]. Our results reinforce this approach and highlight the importance of protocolized, timely HWSC administration.

Despite these promising findings, the primary studies included in this meta-analysis have limitations. Outcomes such as HLOS and time to symptom resolution showed considerable variability due to inconsistencies in study designs and methodologies. Furthermore, incomplete reporting of secondary outcomes, particularly complication rates and time to surgery, in many studies prevented robust conclusions on these parameters. The general absence of subgroup analyses in the original studies also made it challenging to identify the patient cohorts most likely to benefit from HWSC, such as those with partial versus complete obstructions [39].

Limitations 

This study has several limitations. First, we observed substantial statistical heterogeneity for the outcomes of HLOS (I² = 91%) and time to resolution of symptoms (I² = 94%). To explore the sources of this heterogeneity, we performed sensitivity analyses. For the time to resolution of symptoms, the removal of the study by Nishi et al. [23] reduced heterogeneity to 0%, identifying it as a key source of variability. The high heterogeneity for HLOS persisted, potentially reflecting underlying clinical diversity such as differing hospital protocols and patient characteristics. To assess publication bias, we generated a funnel plot for the outcome of operative rates (which included a sufficient number of studies), which showed visual symmetry, suggesting a low risk of such bias.

Second, the synthesis of some outcomes was constrained as several studies were excluded due to missing data (e.g., medians, means, or standard deviations). The scope of our analysis was also limited by the lack of reported data on secondary outcomes like complication rates and time to surgery, which were only available in one or two studies. Additionally, some studies had either short or unreported follow-up periods, preventing an assessment of long-term outcomes such as ASBO recurrence. Finally, the absence of gender distribution data in two studies may affect the precision of our findings [39,40].

Recommendations for Future Research

Based on our findings, we recommend that future research focus on identifying the optimal timing and dosage of HWSC. Developing standardized administration protocols would help reduce outcome variability and improve the generalizability of results [39]. Studies with long-term follow-up are necessary to assess ASBO recurrence and patient-reported outcomes. Future RCTs should prioritize patient stratification to identify those who derive the greatest benefit from HWSC, particularly considering obstruction severity and comorbidities [39].

It is recommended that this review be updated periodically to incorporate new evidence. Future meta-analyses should investigate the causes of heterogeneity through subgroup analyses [13]. International collaboration is encouraged to overcome language barriers and improve accessibility, potentially through multilingual reporting tools and shared global registries for ASBO outcomes. Such efforts are crucial to validate these findings and refine future treatment guidelines.

## Conclusions

Our study aimed to measure the effectiveness of an HWSC (Gastrografin) for ASBO, compared with that of traditional nonoperative methods. The main findings indicated a shorter HLOS and lower operative rate. However, there is a scarcity of data on complications and time to resolution, with substantial variability in patient populations and treatment protocols. Future studies should focus on these factors to enhance quality.

## Appendices

PubMed search strategy

("water-soluble contrast" OR "hyperosmolar contrast" OR "Gastrografin") AND ("small bowel obstruction" OR "intestinal obstruction") No time limits were applied, but the search was restricted to studies published in the English language.

Web of Science search strategy

("water-soluble contrast" OR "hyperosmolar contrast" OR "Gastrografin") AND ("small bowel obstruction" OR "intestinal obstruction") No time limits were applied, but the search was restricted to studies published in the English language.

Google Scholar search strategy

("water-soluble contrast" OR "hyperosmolar contrast" OR "Gastrografin") AND ("small bowel obstruction" OR "intestinal obstruction") No time limits were applied, but the search was restricted to studies published in the English language.

## References

[1] Ten Broek RP, Krielen P, Di Saverio S (2018). Bologna guidelines for diagnosis and management of adhesive small bowel obstruction (ASBO): 2017 update of the evidence-based guidelines from the world society of emergency surgery ASBO working group. World J Emerg Surg.

[2] Aka AA, Wright JP, DeBeche-Adams T (2021). Small bowel obstruction. Clin Colon Rectal Surg.

[3] Chen SC, Chang KJ, Lee PH, Wang SM, Chen KM, Lin FY (1999). Oral urografin in postoperative small bowel obstruction. World J Surg.

[4] Chen SC, Lin FY, Lee PH, Yu SC, Wang SM, Chang KJ (1998). Water-soluble contrast study predicts the need for early surgery in adhesive small bowel obstruction. Br J Surg.

[5] Feuer D, Shepherd J (2002). A review of the management of small bowel obstruction. Ann R Coll Surg Engl.

[6] Assalia A, Kopelman D, Bahous H, Klein Y, Hashmonai M (1997). Gastrografin for mechanical partial, small bowel obstruction due to adhesions [article in Hebrew]. Harefuah.

[7] Feigin E, Seror D, Szold A (1996). Water-soluble contrast material has no therapeutic effect on postoperative small-bowel obstruction: results of a prospective, randomized clinical trial. Am J Surg.

[8] Assalia A, Schein M, Kopelman D, Hirshberg A, Hashmonai M (1994). Therapeutic effect of oral Gastrografin in adhesive, partial small-bowel obstruction: a prospective randomized trial. Surgery.

[9] Scotté M, Mauvais F, Bubenheim M (2017). Use of water-soluble contrast medium (gastrografin) does not decrease the need for operative intervention nor the duration of hospital stay in uncomplicated acute adhesive small bowel obstruction? A multicenter, randomized, clinical trial (Adhesive Small Bowel Obstruction Study) and systematic review. Surgery.

[10] Burge J, Abbas SM, Roadley G, Donald J, Connolly A, Bissett IP, Hill AG (2005). Randomized controlled trial of Gastrografin in adhesive small bowel obstruction. ANZ J Surg.

[11] Moher D, Liberati A, Tetzlaff J, Altman DG (2009). Preferred reporting items for systematic reviews and meta-analyses: the PRISMA statement. PLoS Med.

[12] Thompson M, Tiwari A, Fu R (2012). A Framework To Facilitate the Use of Systematic Reviews and Meta-Analyses in the Design of Primary Research Studies. https://www.ncbi.nlm.nih.gov/books/NBK83621/.

[13] Page MJ, McKenzie JE, Bossuyt PM (2021). The PRISMA 2020 statement: an updated guideline for reporting systematic reviews. Syst Rev.

[14] Higgins JP, Altman DG, Gøtzsche PC (2011). The Cochrane Collaboration's tool for assessing risk of bias in randomised trials. BMJ.

[15] Biondo S, Parés D, Mora L, Martí Ragué J, Kreisler E, Jaurrieta E (2003). Randomized clinical study of Gastrografin administration in patients with adhesive small bowel obstruction. Br J Surg.

[16] Di Saverio S, Catena F, Ansaloni L, Gavioli M, Valentino M, Pinna AD (2008). Water-soluble contrast medium (gastrografin) value in adhesive small intestine obstruction (ASIO): a prospective, randomized, controlled, clinical trial. World J Surg.

[17] Kumar P, Kaman L, Singh G, Singh R (2009). Therapeutic role of oral water soluble iodinated contrast agent in postoperative small bowel obstruction. Singapore Med J.

[18] Rahmani N, Mohammadpour RA, Khoshnood P, Ahmadi A, Assadpour S (2013). Prospective evaluation of oral gastrografin(®) in the management of postoperative adhesive small bowel obstruction. Indian J Surg.

[19] Haule C, Ongom PA, Kimuli T (2013). Efficacy of Gastrografin(®) Compared with Standard Conservative Treatment in Management of Adhesive Small Bowel Obstruction at Mulago National Referral Hospital. J Clin Trials.

[20] Chhabra A, Kumar A, Upadhyay S (2017). Therapeutic role of Gastrograffin in the management of post-operative adhesive small bowel obstruction: a randomized trial. International Journal of Contemporary Medical Research.

[21] Khorshidi HR, Majidi P, Pirdehghan A (2019). Therapeutic effect of gastrografin and predictors of operative intervention in patients with adhesive small bowel obstruction: A randomized controlled study. Turk J Surg.

[22] Katano T, Shimura T, Nishie H (2020). The first management using intubation of a nasogastric tube with Gastrografin enterography or long tube for non-strangulated acute small bowel obstruction: a multicenter, randomized controlled trial. J Gastroenterol.

[23] Nishie H, Shimura T, Katano T (2022). Long-term outcomes of nasogastric tube with Gastrografin for adhesive small bowel obstruction. J Gastroenterol Hepatol.

[24] Lee JF, Meng WC, Leung KL, Yu SC, Poon CM, Lau WY (2004). Water soluble contrast follow‐through in the management of adhesive small bowel obstruction: A prospective randomized trial. Surg Pract.

[25] Nangare N, Nagur B, Biradar S (2016). A prospective study on the Gastrografin in the management of adhesive small bowel obstruction. J Evol Med Dent.

[26] Boutros C, Espat NJ (2010). Further uses of gastrografin in adhesive small bowel obstruction: are we close to a definitive answer. J Surg Res.

[27] Fevang BT, Jensen D, Fevang J (2000). Upper gastrointestinal contrast study in the management of small bowel obstruction--a prospective randomised study. Eur J Surg.

[28] Zorbas KA, Velanovich V, Karachristos A (2020). 1047 adverse outcomes associated with combined liver and rectal resections for synchronous rectal adenocarcinoma and hepatic metastasis. Gastroenterology.

[29] Bogusevicius A, Maleckas A, Pundzius J, Skaudickas D (2002). Prospective randomised trial of computer-aided diagnosis and contrast radiography in acute small bowel obstruction. Eur J Surg.

[30] Zhang Y, Gao Y, Ma Q (2006). Randomised clinical trial investigating the effects of combined administration of octreotide and methylglucamine diatrizoate in the older persons with adhesive small bowel obstruction. Dig Liver Dis.

[31] Anderson CA, Humphrey WT (1997). Contrast radiography in small bowel obstruction: a prospective, randomized trial. Mil Med.

[32] Chen SC, Yen ZS, Lee CC (2005). Nonsurgical management of partial adhesive small-bowel obstruction with oral therapy: a randomized controlled trial. CMAJ.

[33] Diaz JJ Jr, Bokhari F, Mowery NT (2008). Guidelines for management of small bowel obstruction. J Trauma.

[34] Vakil R, Kalra S, Raul S (2025). Role of water-soluble contrast study in adhesive small bowel obstruction: A randomized controlled study. Indian J Surg.

[35] Stordahl A, Laerum F, Gjølberg T, Enge I (1988). Water-soluble contrast media in radiography of small bowel obstruction. Comparison of ionic and non-ionic contrast media. Acta Radiol.

[36] Biondo S, Miquel J, Espin-Basany E (2016). A double-blinded randomized clinical study on the therapeutic effect of Gastrografin in prolonged postoperative ileus after elective colorectal surgery. World J Surg.

[37] Chen JH, Hsieh CB, Chao PC, Liu HD, Chen CJ, Liu YC, Yu JC (2005). Effect of water-soluble contrast in colorectal surgery: a prospective randomized trial. World J Gastroenterol.

[38] Choi HK, Chu KW, Law WL (2002). Therapeutic value of gastrografin in adhesive small bowel obstruction after unsuccessful conservative treatment: a prospective randomized trial. Ann Surg.

[39] Klingbeil KD, Wu JX, Osuna-Garcia A, Livingston EH (2022). The effect of hyperosmolar water-soluble contrast for the management of adhesive small bowel obstruction: a systematic review and meta-analysis. Ann Surg.

[40] Elsolh B, Nguyen MA, Berger FH (2022). Water-soluble contrast in the management of adhesive small-bowel obstruction: a Canadian centre's experience with guideline development and implementation. Can J Surg.

